# Characterization of a novel litchi R2R3-MYB transcription factor that involves in anthocyanin biosynthesis and tissue acidification

**DOI:** 10.1186/s12870-019-1658-5

**Published:** 2019-02-07

**Authors:** Biao Lai, Li-Na Du, Bing Hu, Dan Wang, Xu-Ming Huang, Jie-Tang Zhao, Hui-Cong Wang, Gui-bing Hu

**Affiliations:** 1 School of Advanced Agriculture and Bioengineering, Yangtze Normal University, Fuling, 408100 China; 2 State Key Laboratory for Conservation and Utilization of Subtropical Agro-Bioresources/ Guangdong Litchi Engineering Research Center, College of Horticulture, South China Agricultural University, Guangzhou, 510642 China

**Keywords:** Anthocyanins, Tissue acidification, MYB, bHLH, *Litchi chinensis*, Tobacco, Petunia

## Abstract

**Background:**

Maturation of litchi (*Litchi chinensis*) fruit is characterized by dramatic changes in pigments in the pericarp and flavor compounds in the aril. Among them, the biosynthesis of anthocyanins is most noticeable. Previous studies showed that LcMYB1 and LcbHLH transcription factors participated in regulating the anthocyanin biosynthesis in litchi. However, the roles of other MYB factors remain unclear.

**Results:**

In this study, we cloned and characterized the function of *LcMYB5*, a novel R2R3-MYB identified from litchi transcriptome. Although *LcMYB5* was constitutively expressed in litchi tissues and its expressions was not correlated with tissue coloration, overexpression of *LcMYB5* resulted in enhanced biosynthesis of anthocyanins in tobacco and petunia concurrent with the up-regulation of their endogenous *bHLHs* and key structural genes in anthocyanin precursor biosynthesis. These results indicate that LcMYB5 is an R2R3 transcriptional factor regulates anthocyanin biosynthesis either by directly activating the expression of key structural genes such as *DFR* or by indirectly up regulating the expressions of endogenous *bHLH* regulators. More interestingly, the pH values in petals and leaves from transgenic lines were significant lower than those in both untransformed tobacco and petunia, indicating LcMYB5 is also associated with pH regulation. The expressions of *LcMYB5* and its bHLH partner *LcbHLH1* were consistent with the expression of putative tissue acidification gene *LcPH1*, and the changes in malic acid provided further evidence for the close relationship between LcMYB5 and tissue acidification.

**Conclusions:**

Taking together, our study indicated that LcMYB5 is involved in not only anthocyanin biosynthesis but also tissue acidification.

**Electronic supplementary material:**

The online version of this article (10.1186/s12870-019-1658-5) contains supplementary material, which is available to authorized users.

## Background

Anthocyanins are main secondary metabolites contribute to the red, blue, and purple colors in flowers and fruits. The regulation of the anthocyanin biosynthesis pathway was well established in plants [1, 2]. Three transcription factor families including MYB, basic helix-loop-helix (bHLH), and WDR (WD40 repeats) involved in anthocyanin biosynthesis regulation have been extensively characterized [1, 3]. Among them, R2R3 MYB transcription factors were known to be the crucial regulator and have been characterized in various fruit crops including grapevine (*Vitis vinifera*) [4], apple (*Malus domestica*) [5], bayberry (*Myrica rubra*) [6], pear (*Pyrus pyrifolia*) [7], blood orange (*Citrus sinensis*) [8], and litchi (*Litchi chinensis*) [9].

The R2R3-MYB family is one of the largest gene families which control many aspects of secondary metabolism, and the identity and fate of plant cells [10, 11]. R2R3-MYB proteins are usually clustered in functionally conserved clades. Among the clades, the *Arabidopsis* subgroup 6 MYBs mainly regulate anthocyanin biosynthesis in diverse plant species [12]. In addition, members from other subgroup such as grape (VvMYB5a and VvMYB5b) and petunia (PH4) have been characterized and are thought to affect the plant coloration by controlling the transcription of structural genes involved in anthocyanin biosynthetic pathway and/or vacuolar acidification [13–15]. In structural, these two MYB5s and PH4 displayed significant difference with strong anthocyanin inducing MYB transcription factors in regard to the absence of KPRPR[S/F]F motif (Motif 6) but presence of C1 motif (Lx3GIDPxTHKPL) and C3 motif (DDxF[S/P]SFL[N/D]SLIN[E/D]) [10]. In litchi, 53 R2R3-MYB transcription factors were identified from the pericarp based on RNA-sequencing [16]. Among them, LcMYB1 interacting with LcbHLH1/3 transcription factors regulates the anthocyanin accumulation in tobacco and probably in litchi [17]. The role of other R2R3-MYBs in pigmentation and anthocyanin biosynthesis remains to be identified.

In addition to the accumulation of anthocyanins in the vacuoles, the color of a tissue depends in part on the pH of the vacuolar lumen for acidity affects the absorption spectrum of anthocyanins. Endomembrane compartments are usually acidified by vacuolar-type electrogenic H^+^-ATP hydrolases (V-ATPase) proton pumps, which was responsible for translocating H^+^ from the cytoplasm into the lumen [18, 19]. A previous study showed that subgroup 6 R2R3-MYB transcription factor MdMYB1/10 controls anthocyanin accumulation [20, 21], but a recent study indicated that MdMYB1/10 was also involved in regulating cell pH partially by regulating vacuolar H^+^-ATPase MdVHA-B1 and MdVHA-B2 in apple fruit [20]. In petunia, a WYKY transcription factor called PH3 is the target of AN11-AN1-PH4 complex, which regulates vacuolar acidification in petunia through regulating the expression of *PH5*, a gene encoding tonoplast H^+^ P3A-ATPase [22, 23]. These results suggest that R2R3 MYBs are involved in vacuolar acidification. Vacuolar acidification is closely related to fruit coloration, flavor and senescence. In litchi, however, the mechanism of vacuolar acidification is still poorly documented.

In this study, we described the molecular characterization of LcMYB5, a R2R3-MYB transcriptional regulator isolated from litchi. The expression patterns of *LcMYB5* during pericarp coloration and aril development were investigated. The interactions of LcMYB5 with two nucleic localization LcbHLHs were assessed using both yeast two hybrid and BiFC assays. Moreover, *LcMYB5* were overexpressed in both tobacco and petunia to uncover its function. We proposed that LcMYB5-LcbHLH1 complex is potentially involved in flavonoid biosynthesis and vacuolar acidification.

## Results

### Isolation and sequence analysis of *LcMYB5*

**Fig. 1:**
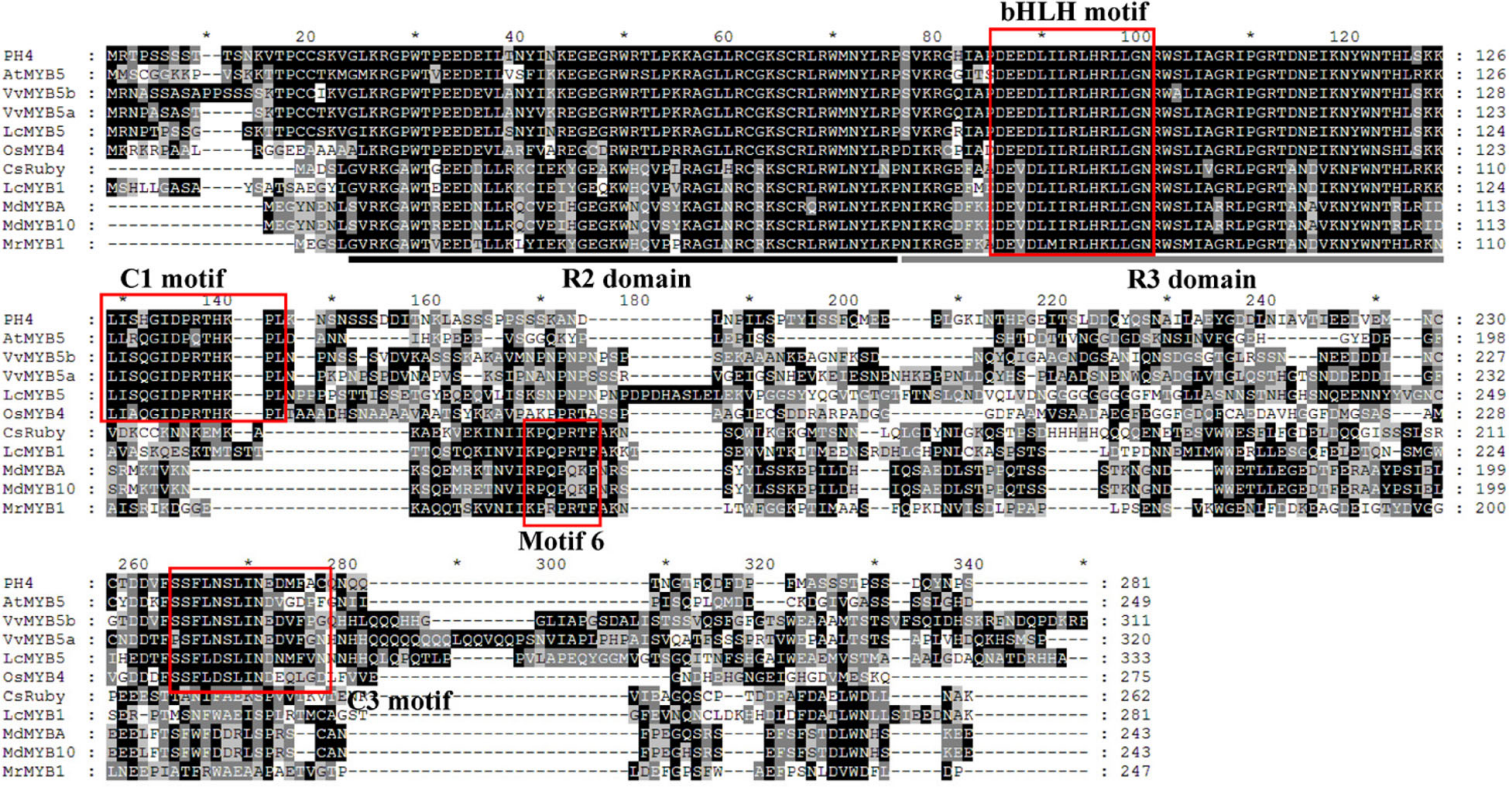
Protein sequence alignment of LcMYB5 and other R2R3-MYB transcription factors from various plant species. Identical residues are shown in black. The R2 and R3 domains are underlined by black and grey lines respectively. Important motifs including bHLH motif, C1 motif, Mitif 6 and C3 motif are marked with red frame

**Fig. 2:**
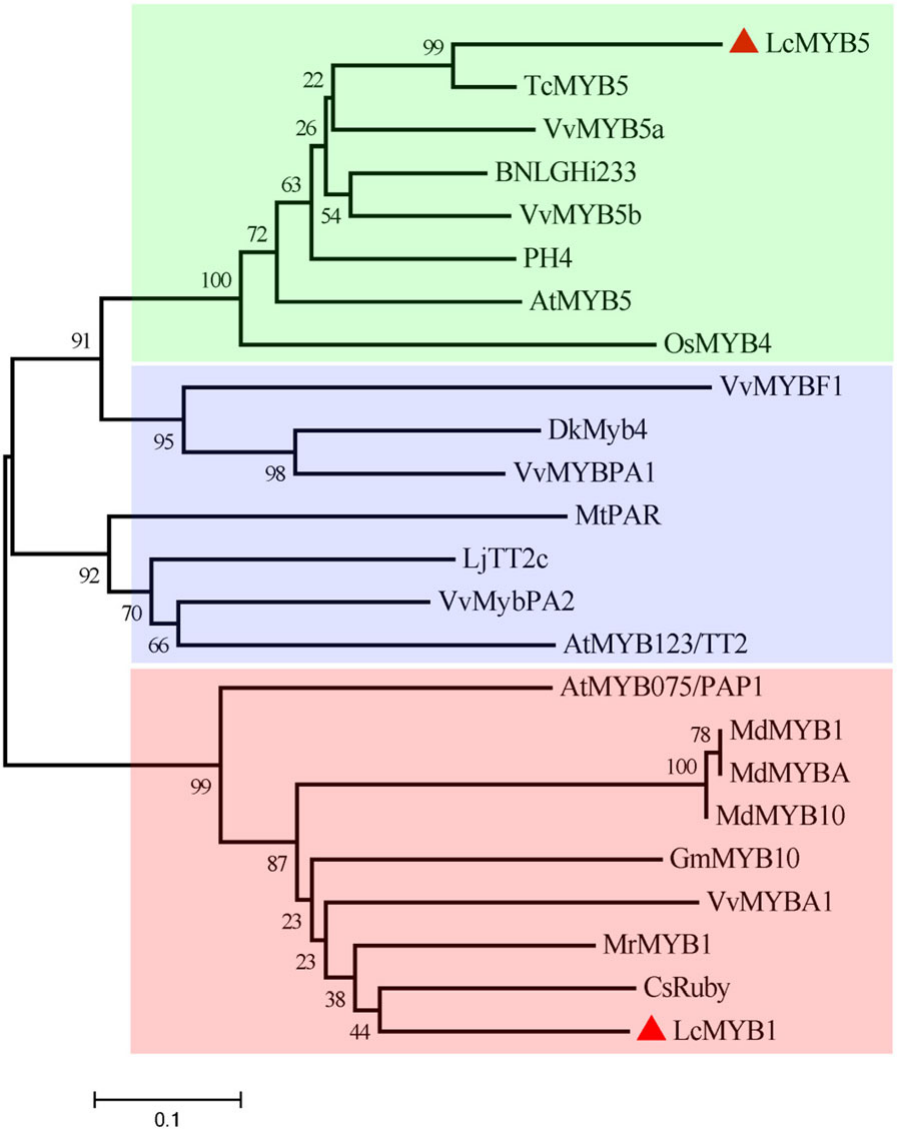
Phylogenetic relationships between LcMYB5, LcMYB1 and R2R3-MYB transcription factors from other plant species. The tree was constructed using MEGA 5, neighboring-joining phylogeny testing, and 1000 bootstrap replicates. The accession number of these proteins (or translated products) are as follows in the GenBank database: MrMYB1, GQ340767; MdMYB1, ABK58136; MdMYB10, DQ267896; MdMYBA, ABB84753.1; GmMYB10, ACM62751.1; CsRuby, AFB73909; MrMYB1, GQ340767; VvMYBA1, BAD18977; LcMYB1, KY302802.1; AtMYB123/TT2, NP_198405.1; DkMyb4, AB503701.1; LjTT2c, AB300035.1; VvMybPA2, NM_001281024.1; VvMYBPA1, NM_001281231.1; MtPAR, HQ337434.1; VvMYBF1, FJ948477.2; AtMYB5, NP_187963.1; TcMYB5, XP_007030073.1; BNLGHi233, AAK19611.1; VvMYB5a, NP_001268108.1; OsMYB4, XP_015644524.1; VvMYB5b, NP_001267854.1; AtMYB075/PAP1, NP_176057.1

Based on our litchi transcriptome [16], one putative R2R3-MYB transcription factor (Unigene 0002103) showed high similarity with AtMYB5, VvMYB5a and VvMYB5b and therefore designated as LcMYB5. The coding region of *LcMYB5 w*as 1002 bp encoding a protein of 333 amino acids which contained typical R2R3 MYB motifs (Fig. 1)*.* In the R3 domain, *LcMYB5* showed an integrated [DE]Lx2[RK]x3Lx6Lx3R motif which could interaction with bHLHs [24]. This suggested that in consistent with LcMYB1 [17], *LcMYB5* may also interact with specific bHLH protein. The KPRPR[S/F]F motif (Motif 6) which is conserved among most MYB transcription factors involved in anthocyanin biosynthesis [10], was not present in LcMYB5. While another two conserved motifs called C1 motif (Lx3GIDPxTHKPL) and C3 motif (DDxF[S/P]SFL[N/D]SLIN[E/D]), which absented in the reported strong anthocyanin inducing MYB transcription factors such as CsRuby, MdMYBA, MdMYB10, MrMYB1 and LcMYB1, was found in MYB5s and PH4 (Fig. 1). A bootstrapped phylogenic tree generated using MEGA5.0 by the neighbor-joining method showed LcMYB5 clustered with VvMYB5a, VvMYB5b and PH4, but not with LcMYB1 (Fig. 2). The amino acid identity over the entire sequence were only 20.3% to LcMYB1, but 37.1% to petunia PH4, 39.8% to grape VvMYB5b, and 40.0% to grape VvMYB5a.

### Subcellular localization and expression patterns of *LcMYB5*

**Fig. 3:**
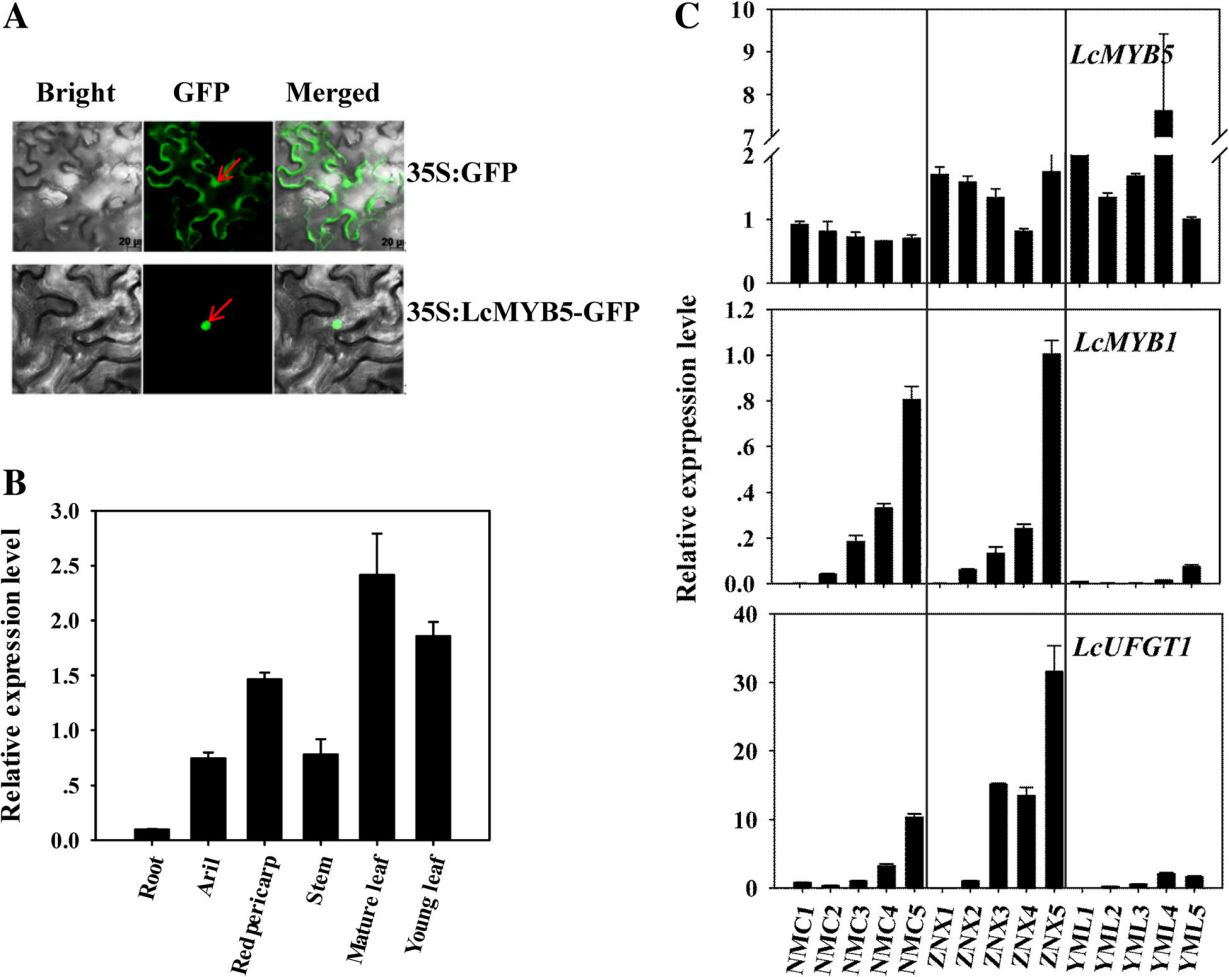
Subcellular localization and expression patterns of *LcMYB5* in relation to *LcMYB1* and *LcUFGT1* expressions. **a** The subcellular localization of LcMYB5. **b** The expression patterns of *LcMYB5* among different litchi tissues. **c** The expression patterns of *LcMYB5*, *LcMYB1* and *LcUFGT1* in different developmental stage pericarps of cultivars Nuomici (NMC), Ziniangxi (ZNX) and Yamulong (YML). The vertical bars represent the standard error of triplicate experiments

MYB transcription factors are always located in the nucleus as a transactivator. To investigate the subcellular localization of LcMYB5, coding sequences of LcMYB5 were fused in frame with *GFP* gene. The LcMYB5-GFP fusion protein was then expressed in *Nicotiana benthamiana* leaves. As showed in Fig. 3a, the fluorescence of LcMYB5 localized exclusively in the nucleus, while that of 35S: GFP control distributed evenly within the cells.

In this study, real-time PCR was used to check the expression patterns of *LcMYB5* in different tissues and in the pericarp during fruit pigmentation in relate to *LcMYB1* and *LcUFGT1*, two key genes determining anthocyanin accumulation in litchi [9, 17]. Inconsistent with *LcMYB1* and *LcUFGT1*, whose expressions paralleled well with pigmentation and anthocyanin accumulation, *LcMYB5* was constitutively expressed in the tissues tested and during pericarp pigmentation (Fig. 3 b-c). Non-red tissues such as mature leaf and the pericarp of ‘YML’ displayed even higher *LcMYB5* expressions than red young leaf and red pericarp. These results implied that *LcMYB5* might not a transcriptional factor involved in anthocyanin biosynthesis in litchi. However, other MYB5 groups such as VvMYB5a, VvMYB5b and PH4 were reported to be involved in tissue pigmentation [13–15]. To confirm the function of *LcMYB5* in anthocyanin biosynthesis, heterologous expression systems were set up.

### Phenotype changes in *LcMYB5* overexpression tobacco lines

**Fig. 4:**
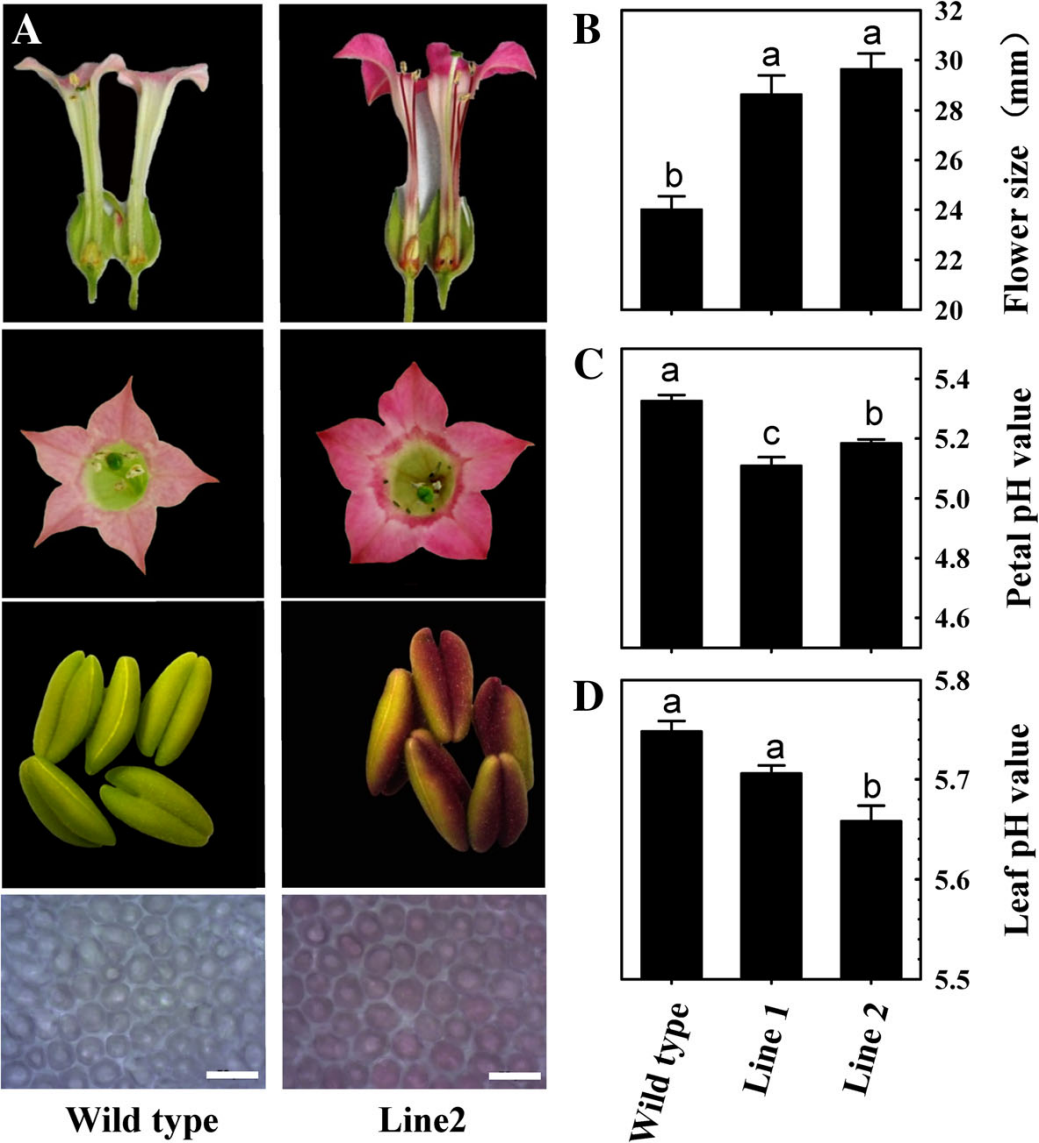
Phenotypes of *LcMYB5* over-expressed tobacco. **a** Images of longitudinal section flower, petal, and anther and microscopic structure of transection petal. **b** Flower size of wild type and *LcMYB5* overex-pressed tobaccos. Petal (**c**) and Leaf (**d**) pH values of wild types and *LcMYB5* overexpressed tobaccos. The vertical bars represent the standard error of triplicate experiments. Different letters on the top of columns indicate significant difference at *p* < 0.05

The *LcMYB5* coding sequence was introduced into the binary pBI121 vector after the CaMV 35S promoter. After *Agrobacterium*-mediated transformation, two transgenic tobacco lines were obtained and significant phenotype changes in flowers were observed (Fig. 4a). In comparing with the flower of wild type, darker petal and pigmented filament, ovary and stamens were noticed. Another obvious change was in the flower size. The flowers of transgenic tobacco lines were significant larger than those of the wild type. The mean corolla diameter of transgenic lines was around 29 mm and that of wild type was around 24 mm (Fig. 4b). Because LcMYB5 was clustered with PH4 transcription factor, a R2R3-MYB involved in vacuolar acidification in petunia, we measured pH values in the petals and leaves in both the transgenic lines and the wild type. In consistent with Quattrocchio et al. [15], significant lower pH values were observed in the petals and leaves of the transgenic lines than those of the wild type (Fig. 4c-d).

### Anthocyanin contents and the expressions of flavonoid biosynthesis genes in transgenic tobacco

**Fig. 5:**
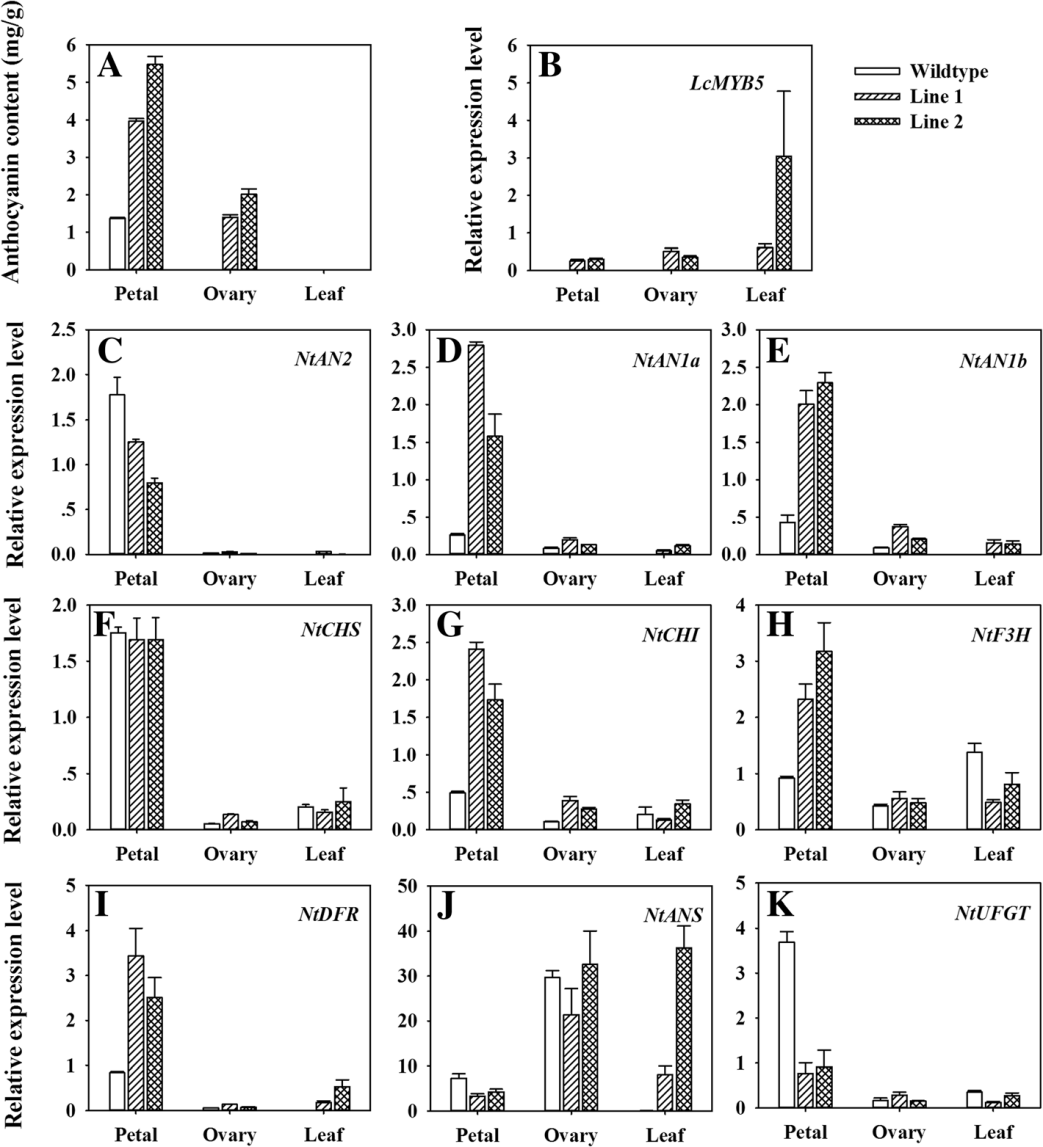
The expressions of anthocyanin biosynthetic pathway structural and regulatory genes in relation to anthocyanin accumulation in wild type and transgenic tobacco lines. **a** Anthocyanin contents in petal, leaf and ovary. **b** The expressions of exogenous *LcMYB5*. **c-k** The expressions of tobacco endogenous regulatory and structural gene in the anthocyanin biosynthetic pathway. The vertical bars represent the standard error of triplicate experiments

Anthocyanin contents in the petal from transgenic lines (4.0–5.5 mg g^− 1^ FW) were about 3–4 times higher than that from wild type (1.4 mg g^− 1^ FW) (Fig. 5a). Anthocyanin accumulation was limited to the reproductive organ in *LcMYB5* over-expressed tobacco lines. In agreement with the non-red phenotype, no anthocyanin was detected in the ovary and leaves of the wild type, while significant amounts of anthocyanins were detected in the ovary of transgenic lines.

Furthermore, the expression levels of anthocyanin biosynthesis genes including *LcMYB5*, three endogenous tobacco anthocyanin biosynthesis regulatory genes, and eight structural genes, were investigated. As shown in Fig. 5b, the expressions of *LcMYB5* were observed in all tissues of the two transgenic lines, but no expression of *LcMYB5* was detected in wild type plants, indicating the successful transformation of *LcMYB5*. Endogenous MYB transcription factor gene *NtAn2* was down-regulated, while the two bHLH transcription factor genes *NtAn1a* and *NtAn1b* were significantly up-regulated in transgenic tobacco lines with the effect being more distinct in petals than in ovaries and leaves (Fig. 5c-e). The expression of *NtAn2* was extremely low or undetectable, while noticeable expressions of *NtAn1a* and *NtAn1b* were observed in the ovaries and leaves in both the transformed lines and the wild type. The structural genes, *NtCHI*, *NtF3H* and *NtDFR* were up-regulated, while *NtANS* and *NtUFGT* were down-regulated in transgenic tobacco petals (Fig. 5f-k). In the ovary, up-regulations of *NtCHS*, *NtCHI* and *NtDFR* were noticed in response to the overexpression of *LcMYB5*. In the leaf, however, significant up-regulation of *NtDFR* and *NtANS* was observed.

### Overexpression of *LcMYB5* induced corolla anthocyanin accumulation and tissue acidification in petunia

**Fig. 6:**
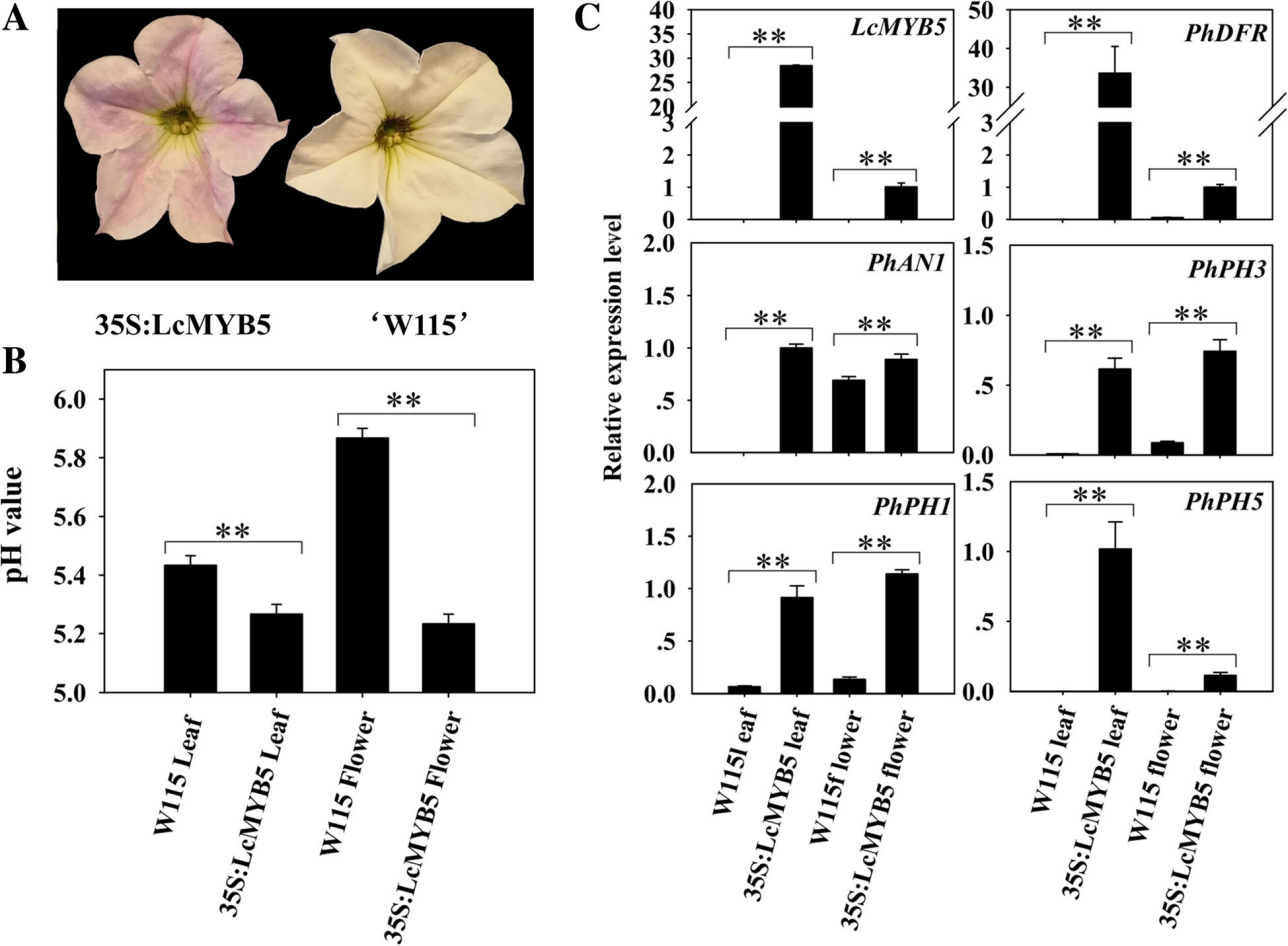
Overexpression of *LcMYB5* in petunia ‘W115’. **a** Image of ‘W115’ and *LcMYB5* over-expressed ‘W115’ flower. **b** pH values in the leaf and flower of ‘W115’ and *LcMYB5* over-expressed ‘W115’. **c** The expressions of exogenous *LcMYB5*, petunia endogenous *PhAN1* (bHLH), key structural gene *PhDFR*, and the reported cell acidication genes in the flower of ‘W115’ and *LcMYB5* over-expressed ‘W115’. The vertical bars represent the standard error of triplicate. **P < 0.01 (t-test, *n* = 3)

To characterize the role of *LcMYB5* in anthocyanin biosynthesis and tissue acidification, 35S:LcMYB5 was stably transformed into petunia W115 (*an2*^−^*an4*^−^). According the results of Quattrocchio, when *AN2* or *AN4* in W115 were over-expressed, the corollas turned blue. In the present study, the corollas of *LcMYB5* over-expressed petunia W115 lines displayed a lavender pink color (Fig. 6a). These results suggested that *LcMYB5* can enhance the anthocyanin biosynthesis in petunia but not so strong as *AN2* and *AN4*, the subgroup 6 MYB members [25]. In consistent with PH4, overexpression of *LcMYB5* resulted in significant decreases in pH value both in flowers and leaves (Fig. 6b). Key anthocyanin biosynthesis gene *PhDFR* was up-regulated in leaves and corollas compared to W115 plants (Fig. 6c). In petunia, *PhAN1* encodes a bHLH transcription factor and *PhPH3* encodes a WRKY transcription factor which regulates two P-ATPase genes, *PhPH5* and *PhPH1* [26]. These genes were all up-regulated in response to the overexpression of *LcMYB5* (Fig. 6c).

### Interaction of LcMYB5 with LcbHLH partners

Previously, we identified two bHLH transcription factors, LcbHLH1 and LcbHLH3, which are nucleic localized and involved in anthocyanin accumulation in tobacco [17]. In this study, the interactions between LcMYB5 and these two LcbHLHs were investigated using Yeast Two-Hybrid (Y2H) assay. Strong transactivation activities in yeast was detected for full-length LcMYB5 fused with the DNA binding domain (DBD). Specific primers were used to amplify the N terminal (1–414 bp) and C terminal (414–1002 bp) sequence of *LcMYB5*, and then these two sequences were ligated into pGBKT7 vectors. The N terminal of LcMYB5 had no transactivation activity in yeast as shown in Fig. 6a. Thus, N terminal of LcMYB5, namely LcMYB5N, was used to investigate the interactions between LcMYB5 and LcbHLHs.

*LcMYB5N* sequence was ligated into pGBKT7 vector and *LcbHLH1* and *LcbHLH3* were cloned into pGADT7 vector for Y2H assay. Yeast cells co-transformed the positive control (pGADT7-T + pGBKT7–53) and *LcMYB5N* with *LcbHLH1*, could grow on SD/ –Leu/−Trp/–His/−Ade medium with the toxic Aureobasidin A, and turned blue when supplied with X-α-Gal (Fig. 6b). However, yeast cells harboring *LcMYB5N* with *LcbHLH3* and the negative controls unable to grow on SD/ –Leu/−Trp/–His/−Ade medium. These results suggested that LcMYB5 interacts with LcbHLH1 but not with LcbHLH3.

To confirm the interaction between LcMYB5 and LcbHLH1, BiFC assays were carried out. LcMYB5 fused N-terminal fragment (NYFP) and *LcbHLH1* fused C-terminal fragment (CYFP) were transiently co-infiltrated to the leaves of *N. benthamiana*. Two days after infiltration, strong YFP fluorescence was noticed in the nucleus of epidermal cells expressing LcMYB5-NYFP and LcbHLH1-CYFP fusion protein, while no YFP fluorescence can be detected either in the cells expressing LcMYB5-NYFP with CYFP or LcbHLH1-CYFP with NYFP (Fig. 6c).

### Expression of *LcMYB5* and *LcbHLH1* during aril development

Above mentioned data indicated that *LcMYB5* could induce flower and leaf acidification in tobacco and petunia and might be associated with the acidification of litchi aril. The contents of malic acid, the predominant acid in the aril of litchi, were determined using HPLC. During early development of litchi aril, increase and high concentrations of malic acids were detected but turned to decrease with aril development toward maturity (Fig. 8a). The expressions of *LcMYB5* and its partner *LcbHLH1* were high during the early stage of aril development, followed by sharp decrease with aril development (Fig. 8 b-c). Their expression patterns were generally consistent with the change pattern of malic acid. These results implied that the LcMYB5-LcbHLH1 complex may participated in the acidification of litchi aril. *PH1* and *PH5* encoding two P-ATPases serve as target of PH4 responsible for petunia petal cell acidification [26]. We searched litchi genome database and found two homolog genes of petunia *PH1*and *PH5* in the aril of litchi, *LcPH1* and *LcPH5*. The alignment and phylogenetic tree of these genes can be found in Additional file 1: Figure S1 and Figure S2. Similar expression patterns of *LcPH1*, *LcMYB5* and *LcbHLH1* were noticed (Fig. 8 b-e). Their expression levels decreased with aril development toward maturity when malic acid declined.

## Discussion

In the present study, a novel R2R3-MYB transcription factor, *LcMYB5*, was obtained by searching the litchi pericarp transcriptomic and genomic database. The coding region of *LcMYB5 w*as 1002 bp encoding a predicted protein of 333 amino acids*. Similar to VvMYB5a*, *VvMYB5b* and *PH4*, genes involved in grape and petunia color regulation [13, 15, 16], LcMYB5 contained bHLH interaction, C1 and C3 motifs (Fig. 1). When compared to LcMYB1 and other key anthocyanin MYB regulators, Motif 6 was absent but C1 and C3 motifs present in LcMYB5. The structural difference suggested a function divergence between these two R2R3-MYB transcription factors.

Phylogenetic analyses showed that LcMYB5 was clearly belonged to a small special cluster (Fig. 2), members in which were reported to have diverse functions in plant, such as color regulation (VvMYB5a, VvMYB5b and PH4), plant cell fate determination (AtMYB5) and chilling tolerance (OsMYB4) [11, 27].

*LcMYB1* is the crucial transcriptional factor in determining anthocyanin biosynthesis in litchi [9]. Unlike *LcMYB1*, which was exclusively expressed in red tissues, *LcMYB5* was found to be expressed in most tissues/organs regardless the endogenous anthocyanin levels (Fig. 3b). And furthermore, the expressions of *LcMYB5* were relatively consistent in the pericarp during fruit coloration when anthocyanin rapidly accumulated and the expressions of *LcMYB1* and *LcUFGT* increased dramatically in two red litchi cultivars ‘NMC’ and ‘ZNX’ (Fig. 3c). These data indicated that *LcMYB5* was constitutively expressed in litchi and that the expression of *LcMYB5* was not correlated well with the fruit coloration. The expression pattern of *LcMYB5* is generally consistent with those of *VvMYB5a* and *VvMYB5b* in grape berry [28]. In studies of Deluc [13, 14], however, demonstrated that *VvMYB5a* and *VvMYB5b* were responsible for regulating proanthocyanin and anthocyanin biosynthesis. These results suggested that the seemingly non correlation between MYB5s and anthocyanin accumulation does not necessarily mean that *MYB5s* is not involved in anthocyanin biosynthesis.

In the present study, overexpression of *LcMYB5* resulted in enhanced biosynthesis of anthocyanins both in tobacco and petunia. The petal color was significantly enhanced and pigmented ovary, filament and stamen were observed in *LcMYB5* overexpression tobacco lines (Fig. 4a). And significantly higher anthocyanins were detected in the petals and ovary in transgenic tobacco lines than in the wild types (Fig. 5a). In petunia assays, lavender pink petal was noticed in *LcMYB5* over-expressed *an2* mutant line W115 as compared to the white petals of untransformed line (Fig. 6a). Although not as strong as that of the PhAN2 wide type (purple blue), the pigmented petals imply that *LcMYB5* enhanced the biosynthesis of anthocyanins, thereby partially complementing the *an2* mutation in the petals. These results indicated that *LcMYB5* is a R2R3 transcriptional factor gene that involved in anthocyanin biosynthesis.

The anthocyanin biosynthesis pathway was the most intensively studied secondary metabolism pathway in plants. It is clear that MYB transcription factor together with bHLH transcription factor control the accumulation of anthocyanins by regulating the transcription of structural genes [29]. Previous studies revealed that the anthocyanin accumulation depended on late structural genes but not early structural genes [6, 30]. *LcMYB1* regulates anthocyanin biosynthesis in tobacco leaves by up-regulating the expression level of *NtDFR*, *NtANS* and *NtUFGT*, the late structural genes [9, 17]. In the present study, significant amount of anthocyanin in the petals of *LcMYB5* overexpression lines was accompanied by dramatically up-regulation of *NtCHI*, *NtF3H* and *NtDFR* (Fig. 5b). A distinct up-regualtion of petunia *DFR* gene was also noticed in both the leaves and petals of *LcMYB5* overexpression lines (Fig. 6b). Unlike *LcMYB1*, which has significant role in activating the gene required for anthocyanidin modification (*LcUFGT*) [9, 17], LcMYB5 enhance anthocyanin synthesis by activating the transcription of genes responsible for biosynthesis of anthocyanin precursor. These results suggested that multiple MYB transcription factors are associated with anthocyanin biosynthesis although they might show differed regulation efficiency and target structural genes.

In consistency with the upregulation of *NtCHI*, *NtF3H* and *NtDFR* (Fig. 5), total flavonoids in both leaves and petals of *LcMYB5* overexpression tobacco lines were significant higher than those in wild types (Additional file 1: Figure S3). In *Arabidopsis*, AtMYB11, AtMYB111 and AtMYB12 have been characterized as flavonol-specific regulators which positively regulate the expression of structural genes in flavonol biosynthesis [31]. Our results suggested that LcMYB5 is a transcription factor involved in up-regulating the expressions of genes responsible for the biosynthesis of anthocyanin precursor (flavonoids) and therefore enhancing anthocyanin accumulation and pigmentation in tobacco and also probably in litchi.

**Fig. 7:**
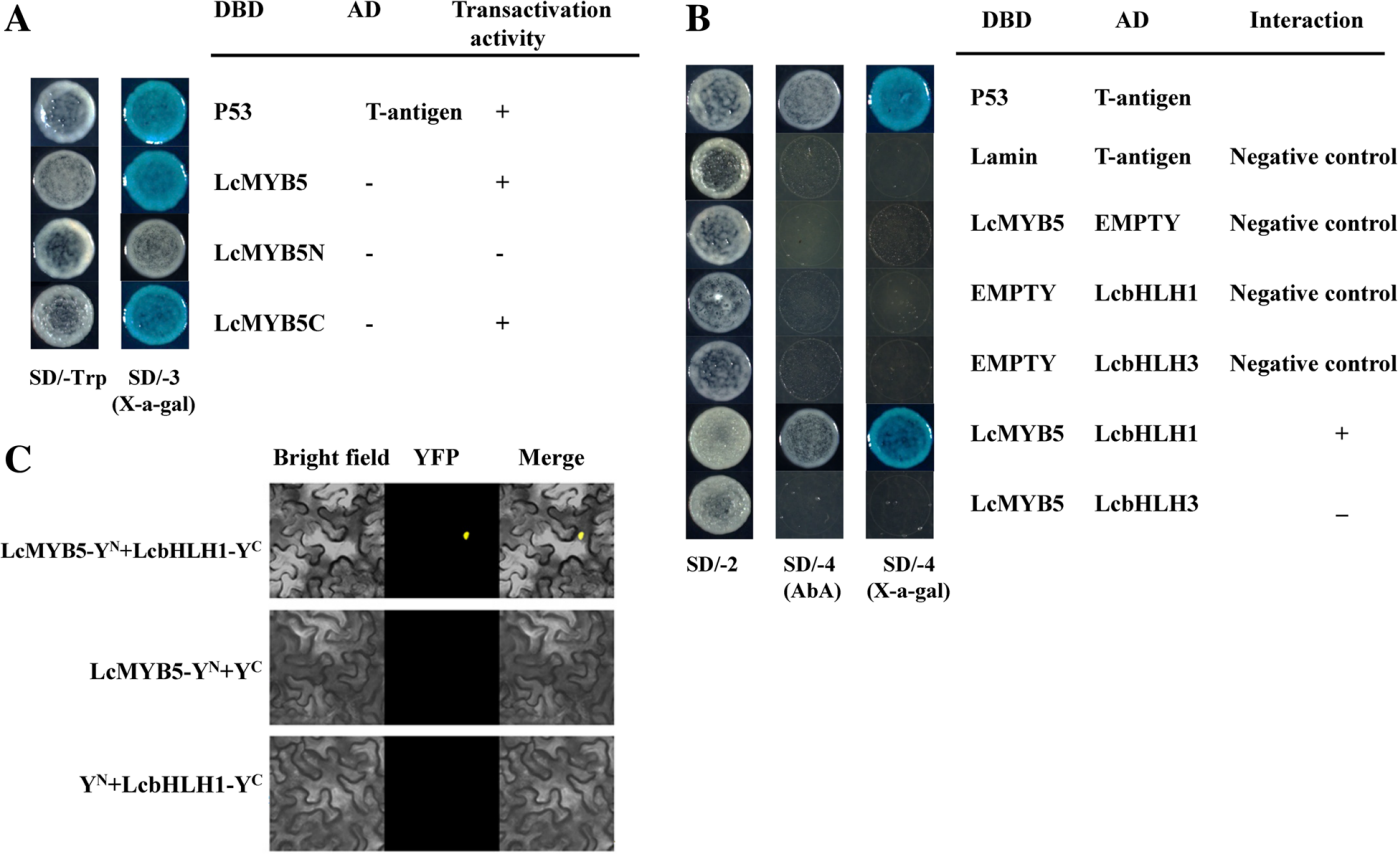
Physical interactions between LcMYB5 and LcbHLH1/3 proteins. **a** Transactivation activity in full-length LcMYB5 and C terminal of LcMYB5, while no transactivation activity in N terminal of LcMYB5 in yeast. **b** All of the constructs together with the positive control (p-53 + T-antigen) and negative control (pGBKT7) were transformed into yeast strain Gold Y2H. Transcription activation was monitored by the detection of yeast growth and an α-galactosidase (α-Gal) assay. **c** Bimolecular fluorescence complementation (BiFC) visualization of the LcMYB5 and LcbHLH1 interaction in transiently co-expressed *N. benthamiana* leaf. The length of the bar indicated in the photographs is 20 μm

Small subfamily bHLH transcription factors function as anthocyanin regulator have been reported in various plant species. *MYB* genes isolated from apple, Chinese bayberry and peach do not induce anthocyanin accumulation when transiently expressed in tobacco leaves without the expression of *bHLH* genes [32–35]. The up-regulation of *NtAn1b* (*bHLH*) in response to *LcMYB1* seems to be crucial for anthocyanin biosynthesis in the tobacco leaves [9]. The anthocyanin content in tobacco leaves increased when *LcMYB1* was co-expressed with *LcbHLH1* or *LcbHLH3* [17]. These results suggested that the regulation of anthocyanin biosynthesis involves multiple bHLHs. Both Y2H and BiFC assays indicated that LcMYB5 interacted with LcHLH1, a transcription factor involved in anthocyanin accumulation (Fig. 7) [17]. Previous dual-luciferase assay demonstrated that the promoter activities of *LcCHS*, *LcCHI*, *LcDFR* and *LcANS* were significantly higher when *LcHLH1* or *LcHLH3* were co-transformed with *LcMYB1* as compared with transformed *LcMYB1* alone [17]. In the present study, tobacco endogenous *bHLH* transcription factors, *NtAn1a* and *NtAn1b,* were significantly up-regulated in all tissues tested, the effect being most obvious in the petals of *LcMYB5* overexpression lines (Fig. 5b). And the expressions of petunia endogenous bHLH regulator, *PhAN1*, were also significantly up-regulated in the leaves and flowers of *LcMYB5* overexpression W115 lines (Fig. 5c). The enhanced effect of LcMYB5 on anthocyanin synthesis in tobacco and petunia might derive from either directly activating the expression level of structural genes like *CHI*, *F3H* and *DFR* and/or indirectly up-regulating the expressions of endogenous *bHLH* regulators.

**Fig. 8:**
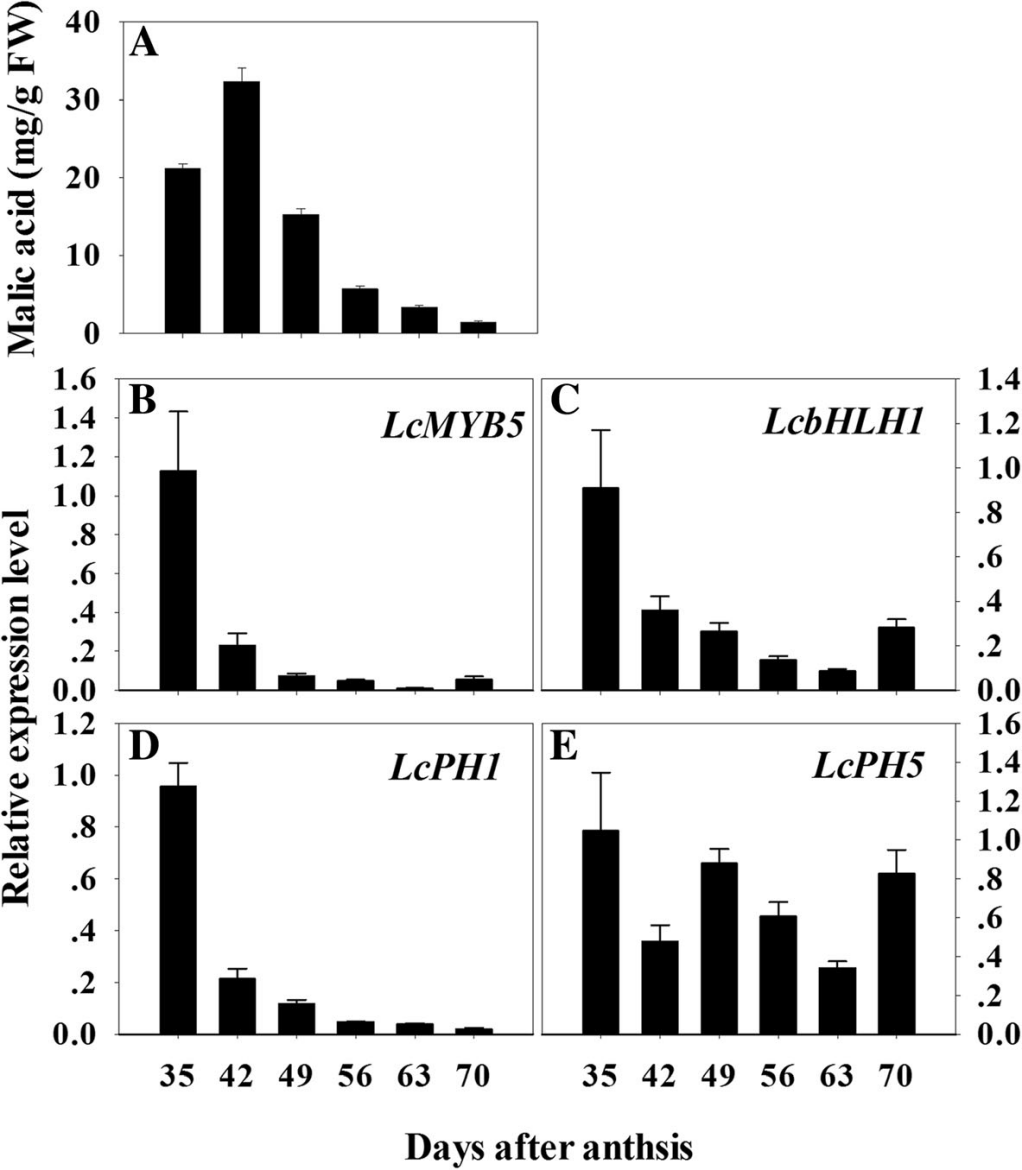
The expression of putative tissue acidification genes in relation to the changes of malic acid in the aril during litchi fruit development. **a** The changes of malic acid contents. **b-e** The expressions of *LcMYB5*, *LcbHLH1*, *LcPH1* and *LcPH5* during aril development. The vertical bars represent the standard error of triplicate

The enhanced petal color might be due to either higher anthocyanins and/or a lower pH value as anthocyanins tend to be scarlet under acid condition. *PH4*, a *R2R3-MYB* gene of petunia, regulates petal color by modifying vacuole acidification instead of the anthocyanin biosynthesis pathway [15]. In the present study, except for enhanced anthocyanin accumulation, a significant decrease in pH value was observed in the petals of transgenic tobacco and petunia (Figs. 4b-d, 6b). In the leaves of these transgenic lines, pH value also significantly reduced compared with untransformed plants. In the aril tissue of litchi, the expression pattern of *LcMYB5* was generally consistent with that of malic acid, the major organic acid in aril (Fig. 8). These results implied that *LcMYB5* is involved not only in anthocyanin biosynthesis but also in cell acidification. Similarly, *VvMYB5a* and *VvMYB5b* could partially restore petunia *an2* mutant plants and also restore petunia *ph 4* mutants by acidification of the vacuole [28].

Regulation of cell acidification was a very complex biological process and pH value in cellular compartments is important for intracellular trafficking of proteins and vesicles and the transport of small molecules, including hormones [36]. In petunia, the expressions of cell acidification related gene *PhPH5* was up-regulated in response to the overexpression of *LcMYB5* in both the petals and the leaves of petunia (Fig. 6c). In the aril of litchi, the expression patterns of *LcMYB5* partners, *LcbHLH1* and *LcPH1,* were paralleled with the expression of *LcMYB5* (Fig. 8). These results implied that LcMYB5 might interact with LcbHLH1 and regulate the key genes involved in cell acidity in litchi. The exact role of *LcMYB5* in regulating tissue pH value in litchi needs to be verified and how it works remained to be uncovered. In the present study, the petal size of the transgenic lines was significantly larger than that of the wild type in paralleling with the cellular acidification (Fig. 4b). Whether the larger petal size in the transgenic lines derived from changes in the cell acidity or other metabolic pathways in response to overexpression of *LcMYB5* needs to be further studied.

## Conclusions

*LcMYB1* is the only previously reported litchi MYB involve in flavonoid biosynthesis regulation. In this study, we cloned and characterized a novel litchi R2R3-MYB transcription factor, *LcMYB5*, which behave distinctively in the induction of anthocyanin biosynthesis in both tobacco and petunia. We found that *LcMYB5* participate in the regulation of anthocyanin accumulation either by directly activating the expressions of key structural genes or by indirectly up regulating the expressions of endogenous *bHLH* regulators. Moreover, our data suggest that other processes such as cell acidficaton may also be controlled by this trancription factor. Although its function has been inferred in heterologous systems, and need to be further confirmed in litchi, our results has nevertheless provided a novel insight into the function of *LcMYB5* and the multiple transcription factors regulated anthocyanin biosynthesis pathway.

## Methods

### Plant materials

Two red litchi cultivars ‘Nuomici’ (‘NMC’) and ‘Ziniangxi’ (‘ZNX’) and one non-red cultivar ‘Yamulong’ (‘YML’) were selected in this work. Trees of cultivars ‘ZNX’ and ‘YML’ were grown in the experimental plantation of Hainan Academy of Agricultural Sciences (Haikou, China), while trees of ‘NMC’ were grown in the experimental orchard of South China Agricultural University (Guangzhou, China). Root, young stems, arils, and young and mature leaves were collected from cultivar ‘ZNX’. Pericarp discs of each cultivar were collected for five times at 5 day intervals before commercial maturity as reflected by aril Brix-acid ratio and were named as sample 1 to 5 in the sequence of maturity. All samples were frozen in liquid nitrogen immediately and then stored in − 80 °C freezer.

Tobacco (*N. tabacum*) ‘K326’ and Petunia (*Petunia hybrida*) ‘W115’ leaf discs were used for *Agrobacterium*-mediated stable transformation [37]. Leaves of *N. benthamiana* were used for subcellular localization and BiFC analysis using infiltration method [17]. Tobacco, petunia and *N. benthamiana* plants were grown in green houses at 28 °C.

### Anthocyanin, malic acid and pH value determination

The contents of anthocyanins in tissues of litchi and tobacco were determined according to our previous study [38]. Malic acid was extracted by grinding 1 g of aril tissue in 5 ml 0.2% (*w*/*v*) HPO3. The supernatant was analyzed by high-performance liquid chromatography (HPLC, Agilent 1200). Detection of malic acid was performed at 210 nm using a diode array detector. A NUCLEODUR C18 column (250 mm × 4.6 mm) was used for separation at 35 °C with 0.2% (w/v) HPO3 at a flow rate of 1 ml min^− 1^. The pH values of petal and leaf extracts were measured directly by grinding the samples in 6 mL of distilled water using a normal pH electrode.

### RNA extraction, gene cloning and sequence analysis

Total RNA was isolated from tissues of litchi, tobacco and petunia using HiPure Plant RNA Kit (Magen, Guangzhou, China). The genomic DNA from total RNA was then digested with TURBO DNAase (Ambion, USA). cDNAs were synthesized from 2 ng total RNA using M-MLV (Invitrogen, USA) according to the manufacturer’s protocol in 20 μl of total volume. The cDNA of red pericarp of cultivar ‘NMC’ was used as the PCR templates to amplify ORF (Open Reading Frame) sequence encoding for LcMYB5 using LcMYB5_F: ATGAGGAACCCAACACCATC and LcMYB5_R: TTATGCATGGTGACGATCCGTAG primer pair. Amplified PCR products were then cloned into cloning vector pTOPO-TA (Aidlab, China) and transformed into JM109 competent cells. Plasmid DNA was isolated from positive *E. coli* cells and sequenced. MUSCLE and MEGA5 were used to perform multiple sequence alignment and phylogenetic tree analysis [39].

### Real-time quantitative PCR

The transcription levels of anthocyanin regulatory and biosynthetic pathway genes in litchi, tobacco and petunia were calculated using real-time PCR using SYBR regent (TaKaRa, Japan) as described previously using an ABI 7500 real-time PCR machine (Applied Biosystems, USA) [9, 23]. The specific primers for gene expression analysis were listed in Additional file 1: Table S1. Relative expression levels of target genes were normalized to the Cp values of housekeeping genes *LcACTIN* and *LcGAPDH* for litchi [40], *NtACTIN* for tobacco and *PhSAND* for petunia. The relative expression levels of the genes were determined using 2^-△△CT^ algorithm [41]. All the above analyses were carried out with three biological replicates.

### Subcellular localization analysis

Sequence coding for LcMYB5 (without the stop codon) was amplified, and then ligated into the pEAQ-GFP vector, which was linearized by restriction enzyme *Age* I, in frame with the GFP (green fluorescent protein) sequence using In-Fusion HD Cloning kit (Clontech, USA) (primers pairs are listed in Additional file 1: Table S2) [42]. The 35S: LcMYB5-GFP fusion construct and the control 35S: GFP vector were transformed into *Agrobacterium tumefaciens* strain GV3101 and then infiltrated into *N. benthamiana* leaves separately. GFP fluorescence signal was observed 2 days after infiltration using the fluorescence microscope (Axio Observer D1, Zeiss). All transient infiltration experiments were repeated at least three times.

### Yeast two-hybrid assay

Yeast (*Saccharomyces cerevisiae*) two-hybrid assays were performed using the Matchmaker GAL4-based two-hybrid system according to the manufacturer’s instructions (Clontech). The coding region of *LcMYB5*, *LcMYB5* N terminal (*LcMYB5N*, 1–417 bp) and *LcMYB5* C terminal (*LcMYB5C*, 418–1002 bp) were ligated into pGADT7 vector to fuse with the activation domain (DNA-AD) (primers pairs are listed in Additional file 1: Table S2). Full length of *LcMYB5* displayed auto transcriptional activation activity in yeast cells. N terminal of *LcMYB5* (LcMYB5N) did not show while C terminal of *LcMYB5* (LcMYB5C) showed auto transcriptional activation activity in yeast cells. Specific bait and prey constructs combinations were then co-transformed into Gold Y2H yeast strain through a lithium acetate method and then yeast cells were plated selected on SD/−Leu/−Trp medium for 3 days. The transformed colonies were then plated on SD/−Leu/−Trp/–His/−Ade medium containing appropriate amount of Aureobasidin A and X-α-Gal at 30 °C for 3 days. The interactions between LcbHLH1/3 and LcMYB5 were determined according to the growth of the yeast cells and the activity of α-galactosidase.

### BiFC assays

Sequence coding (without the stop codon) for LcMYB5 was amplified (primers pairs are listed in Additional file 1: Table S2), and subcloned into the pEAQ-NYFP linearizated by *Age* I using In-Fusion method to make LcMYB5-NYFP fusion protein. pEAQ-LcbHLH1-CYFP and pEAQ-LcbHLH3-CYFP were from our previous publication [17]. All constructs were then transformed into *A. tumefaciens* strain GV3101 and then infiltrated into *N. benthamiana* leaves. YFP fluorescence was observed two days after infiltration under the fluorescence microscope (Axio Observer D1, Zeiss). Expressions of genes fuse to NYFP or CYFP alone in the leaves were used as negative controls. All transient infiltration experiments were repeated at least three times.

## Additional file

Additional file 1: **Figure S1.** The alignment of the amino acid sequences. (A) Sequences of PhPH1 and LcPH1. (B) Sequences of PhPH5 and LcPH5. **Figure S2.** Phylogenetic relationships of LcPH1 and LcPH5 with homolog proteins from other species (Sequences are from Li Y, Provenzano S, Bliek M, et al. Evolution of tonoplast P-ATPase transporters involved in vacuolar acidification. New Phytologist, 2016, 211(3):1092–1107.) **Figure S3.** Total flavonoid contents of leaf and petal in wild types and LcMYB5 over-expressed tobacco lines. **Table S1.** Primers used for real-time PCR. **Table S2.** Primers used in protein interaction experiments. (DOCX 1626 kb)

## Abbreviations

ANS: Anthocyanidin synthase
CHI: Chalcone isomerase
CHS: Chalcone synthase
DFR: Dihydroflavonol reductase
UFGT: UDP-flavonoid glucosyltransferase
X-α-Gal: 5-bromo-4-chloro-3-indoxyl a-D-galactoside
