# Supplementary material for: Characterization of a novel litchi R2R3-MYB transcription factor that involves in anthocyanin biosynthesis and tissue acidification
Source: BMC Plant Biol. 2019 Feb 7;19:62. doi: 10.1186/s12870-019-1658-5 (PMC6367832; doi:10.1186/s12870-019-1658-5)
Supplement: Supplementary file 1 — Figure S1. The alignment of the amino acid sequences. (A) Sequences of PhPH1 and LcPH1. (B) Sequences of PhPH5 and LcPH5. Figure S2. Phylogenetic relationships of LcPH1 and LcPH5 with homolog proteins from other species (Sequences are from Li Y, Provenzano S, Bliek M, et al. Evolution of tonoplast P-ATPase transporters involved in vacuolar acidification. New Phytologist, 2016, 211(3):1092–1107.) Figure S3. Total flavonoid contents of leaf and petal in wild types and LcMYB5 over-expressed tobacco lines. Table S1. Primers used for real-time PCR. Table S2. Primers used in protein interaction experiments. (DOCX 1626 kb) [file 12870_2019_1658_MOESM1_ESM.docx]

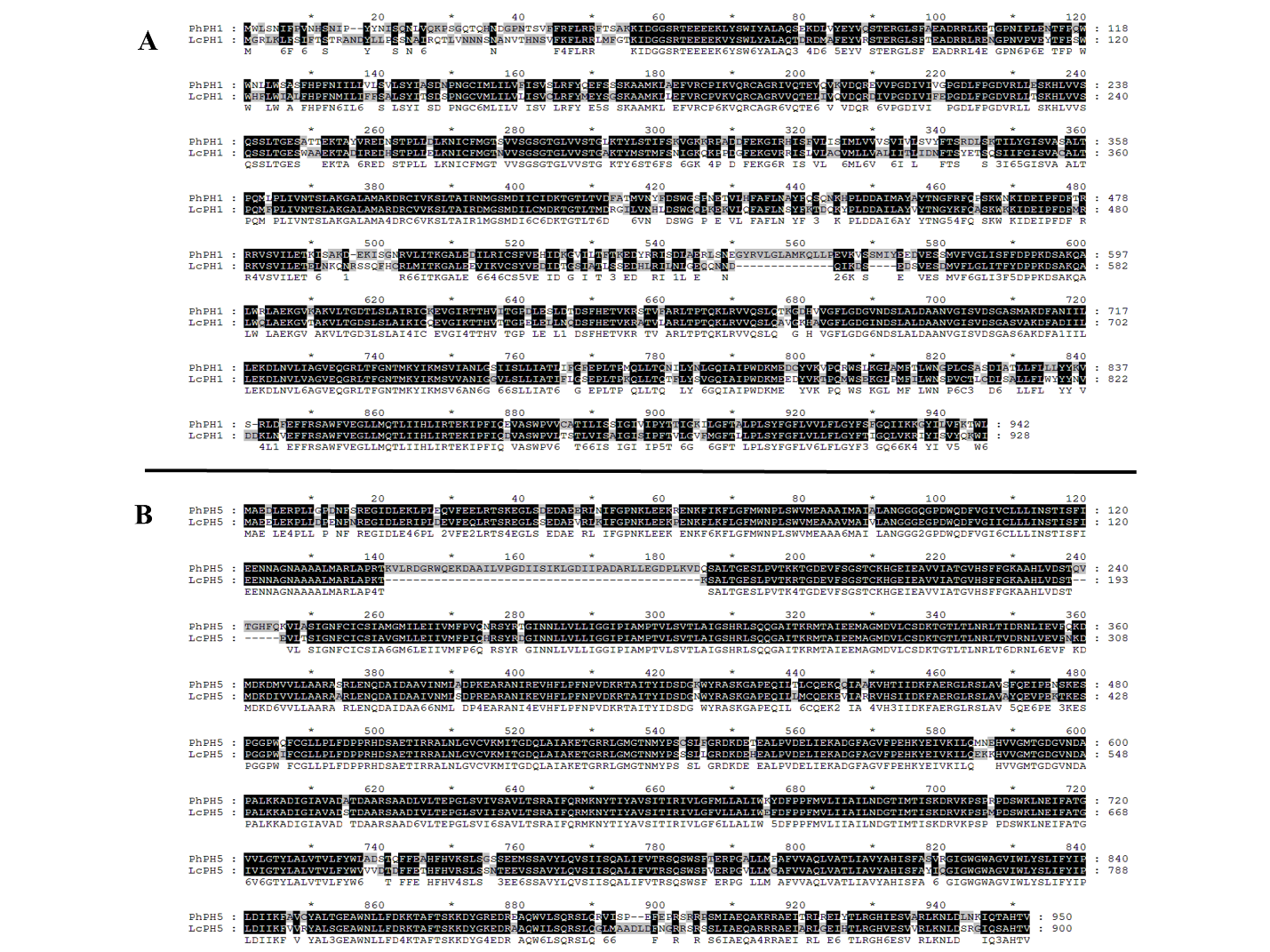


**Figure S1** Alignment of the amino acid sequences of PhPH1 and LcPH1 (A), PhPH5 and LcPH5 (B)

******Figure S2** Phylogenetic relationships of LcPH1 and LcPH5 with homolog proteins from other species (Sequences are from Li Y, Provenzano S, Bliek M, et al. Evolution of tonoplast P-ATPase transporters involved in vacuolar acidification[J]. New Phytologist, 2016, 211(3):1092-1107.).

**Figure S3** Total flavonoid contents of leaf and petal in wild types and LcMYB5 over-expressed tobacco lines.

**Table S1.** Primers used for real-time PCR

| Name | Forward primer  (5' to 3') | | Reverse primer  (5'to 3') |
| --- | --- | --- | --- |
| *LcMYB5* | | CGGTAACAGATGGTCTTTGAT | TGTAGTAGAAGGCGGTGGTG |
| *LcACTIN* | | ACCGTATGAGCAAGGAAATCACTG | TCGTCGTACTCACCCTTTGAAATC |
| *LcGAPDH* | | GATACAGTTCCCGTGTTGTTGAC | CATAAAGACACATAACACCACACTC |
| *LcbHLH1* | | CGAAGCCTGTTTGCTCTG | ATTCATCACCTCCATCCTGA |
| *LcPH1* | | CAAATCAAAGACAGCGAGGA | TTATTGCCAGGGAGAGTGAA |
| *LcPH5* | | CAAAGGAGACAGGCAGACGA | TCAGGGAATACACCAGCAAA |
| *PhPH1* | | AGTCCAATATTCAATGTGGTTATC | TCAGTT CTCGACCCTCCATC |
| *PhPH3* | | GTTTAATGCAGGTACTATGACAGGA | CATCTTTGTCAAGTACTTCACTAGT |
| *PhPH5* | | tagcaatcctaaatgatggcact | caactatcaggtcttggagatgg |
| *PhDFR* | | ACCTATGGATTTCGAGTCCAAAGA | CACATGATTCAATGATGCTTAGCAT |
| *PhAN1* | | CTTCCAATCCCACCACATTT | TCGTCCTCCTCATCCTCATC |
| *PhSAND* | | CTTACGACGAGTTCAGATGCC | TAAGTCCTCAACACGCATGC |

**Table S2.** Primers used in protein interaction experiments

| Name | Sequences (5＇-3＇) | Function |
| --- | --- | --- |
| LcMYB5-GFP_F | CAAATTCGCGACCGGTATGAGGAACCCAACACCATC | Fusion GFP |
| LcMYB5-GFP_R | TGCTAGCCATACCGGTTGCATGGTGACGATCCGTAG |  |
| LcMYB5-BD_F | CATGGAGGCCGAATTCATGAGGAACCCAACACCATCTT | Yest two hybrid |
| LcMYB5-BD_R | GCAGGTCGACGGATCCTTATGCATGGTGACGATCCG |  |
| N-LcMYB5-BD_R | GCAGGTCGACGGATCCTTACAAAGGTTTGTGTGTTCTCG |  |
| C-LcMYB5-BD_F | CATGGAGGCCGAATTCATGAACCCACCACCGCCTT |  |
| LcMYB5-NYFP_F | CAAATTCGCGACCGGTATGAGGAACCCAACACCATC | BiFC |
| LcMYB5-NYFP_R | TGCTCACCATACCGGTTGCATGGTGACGATCCGTAG |  |
